# 5*α*-Epoxyalantolactone from *Inula macrophylla* attenuates cognitive deficits in scopolamine-induced Alzheimer’s disease mice model

**DOI:** 10.1007/s13659-024-00462-y

**Published:** 2024-07-02

**Authors:** Rui Ma, Xu-Yao Feng, Jiang-Jiang Tang, Wei Ha, Yan-Ping Shi

**Affiliations:** 1 CAS Key Laboratory of Chemistry of Northwestern Plant Resources, Key Laboratory for Natural Medicines of Gansu Province, Lanzhou Institute of Chemical Physics, Chinese Academy of Sciences (CAS), Lanzhou, 730000 People’s Republic of China; 2 Shaanxi Key Laboratory of Natural Products & Chemical Biology, College of Chemistry & Pharmacy, Northwest A&F University, No. 3 Taicheng Road, Yangling, 712100 Shaanxi China

**Keywords:** Alzheimer’s disease, 5*α*-Epoxyalantolactone (5*α*-EAL), Anti-neuroinflammation, Attenuates cognitive deficits

## Abstract

Alzheimer’s disease (AD) is a complex neurodegenerative condition. 5*α*-epoxyalantolactone (5*α*-EAL), a eudesmane-type sesquiterpene isolated from the herb of *Inula macrophylla*, has various pharmacological effects. This work supposed to investigate the improved impact of 5*α*-EAL on cognitive impairment. 5*α*-EAL inhibited the generation of nitric oxide (NO) in BV-2 cells stimulated with lipopolysaccharide (LPS) with an EC_50_ of 6.2 μM. 5*α*-EAL significantly reduced the production of prostaglandin E2 (PGE_2_) and tumor necrosis factor-α (TNF-α), while also inhibiting the production of cyclooxygenase-2 (COX-2) and inducible nitric oxide synthase (iNOS) proteins. The ability of 5*α*-EAL to penetrate the blood–brain barrier (BBB) was confirmed via a parallel artificial membrane permeation assay. Scopolamine (SCOP)-induced AD mice model was employed to assess the improved impacts of 5*α*-EAL on cognitive impairment in vivo. After the mice were pretreated with 5*α*-EAL (10 and 30 mg/kg per day, *i.p.*) for 21 days, the behavioral experiments indicated that the administration of the 5*α*-EAL could alleviate the cognitive and memory impairments. 5*α*-EAL significantly reduced the AChE activity in the brain of SCOP-induced AD mice. In summary, these findings highlight the beneficial effects of the natural product 5*α*-EAL as a potential bioactive compound for attenuating cognitive deficits in AD due to its pharmacological profile.

**Graphical Abstract:**

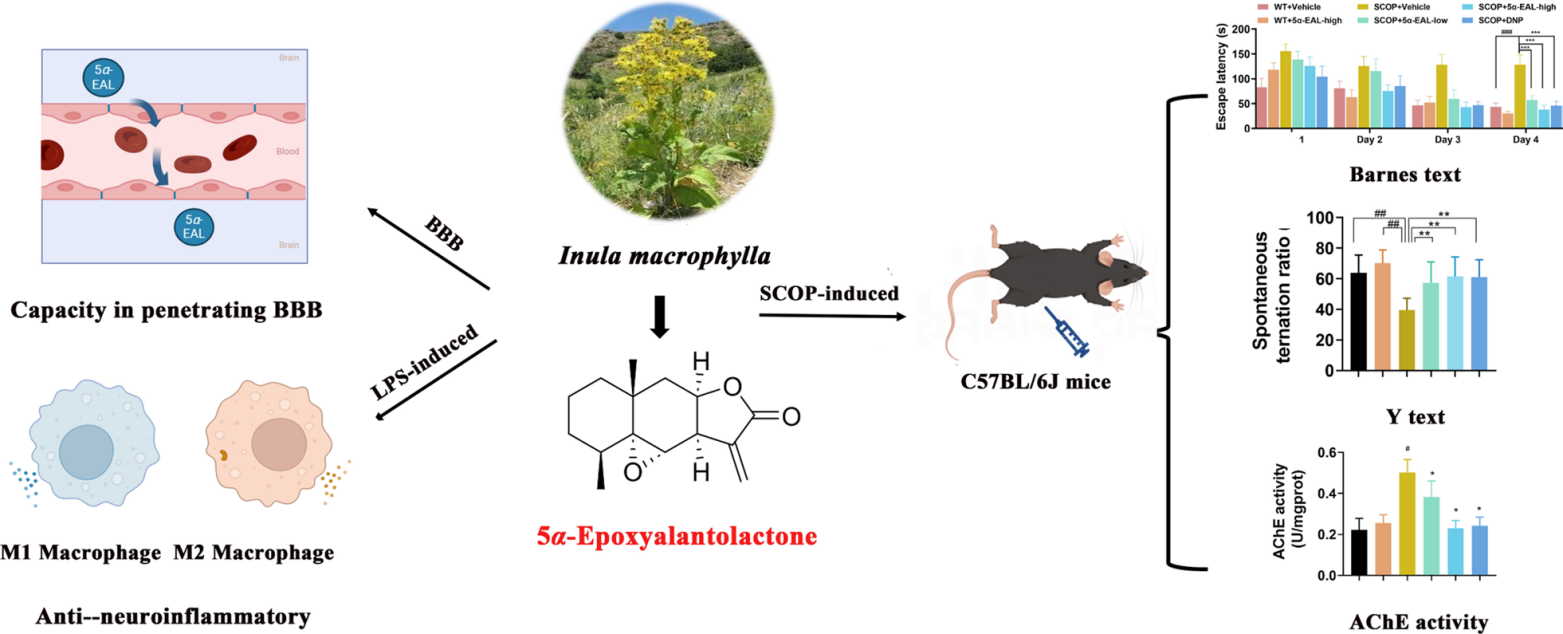

**Supplementary Information:**

The online version contains supplementary material available at 10.1007/s13659-024-00462-y.

## Introduction

Alzheimer’s disease (AD) is a multifactorial neurodegenerative condition and the most common type of dementia, that manifests itself as memory impairment and cognitive dysfunction. AD is characterized by a disability in performing daily living activities, and is often accompanied by a range of mental symptoms and behavioral disorders [1, 2]. As the worldwide population continues to grow older, AD patients are expected to reach 152 million by 2050. The economic impact of AD is expected to cost the global economy over 1 trillion dollars [3]. Improvement of subjective cognitive deficits might be a valuable therapeutic strategy in the management of dementia, which delays or prevents the progression of cognitive impairment to AD [4].

The diversity of scaffolds and the structural complexity found in natural products have made a crucial contribution throughout the history of the evolution of drugs [5], which has proved to be a treasure of drug treatment for AD. For example, galantamine and huperzine A, natural alkaloids cholinesterase inhibitors, were extensively utilized in the treatment of AD [6, 7]. The genus *Inula*, from the Asteraceae family, has been commonly employed in traditional medicine and has recently attracted increasing attention due to its application in neurodegenerative conditions [8–11]. Eudesmane-type sesquiterpene lactones (SLs) containing an α-methylene-γ-lactone moiety as the representative genus *Inula* secondary metabolites, have attracted increasing attention for their pharmacological activities against central nervous system-related disorders [6, 12, 13]. For example, the alantolactone and isoalantolactone, eudesmane-type SLs isolated from *Inula helenium*, which has neuroprotective effects by significantly attenuating Aβ25–35 (10 μM) demonstrated cytotoxicity in neuronal cells derived from the mice brain and preventing ROS-mediated cognitive impairment through the Nrf2 signaling pathway [14]. Hence, the eudesmane-type SLs in genus *Inula* exhibit significant potential as lead compounds for further exploration of preventative and therapeutic agents of AD.

*Inula macrophylla* is mainly restricted to northern temperate regions. In Central Asia, *Inula macrophylla* is traditionally used as a remedy for lung diseases, gastric ulcers, bronchitis, diabetes, and intestinal ulcers in traditional medicine [14, 15]. Nevertheless, the effects of *Inula macrophylla* on AD therapy have been less explored currently. Our previous research screened secondary metabolites isolated from *Inula macrophylla* by an anti-neuroinflammatory cell model. 5*α*-Epoxyalantolactone (5*α*-EAL) was identified as an eudesmane-type SLs that exhibited excellent inhibition of NO production in LPS-stimulated BV-2 cells [16]. In this work, the effects of 5*α*-EAL on the production of nitric oxide (NO), prostaglandin E_2_ (PGE_2_), tumor necrosis factor-α (TNF-α), and interleukin 10 (IL-10), as well as the protein expression of cyclooxygenase-2 (COX-2) and nitric oxide synthase (iNOS) were thoroughly investigated using LPS-stimulated BV-2 microglial cell model. Furthermore, the potential improvement in cognitive deficits in SCOP-induced AD mice resulting from 5*α*-EAL was also evaluated.

## Results and discussion

### Chemistry

**Fig. 1:**
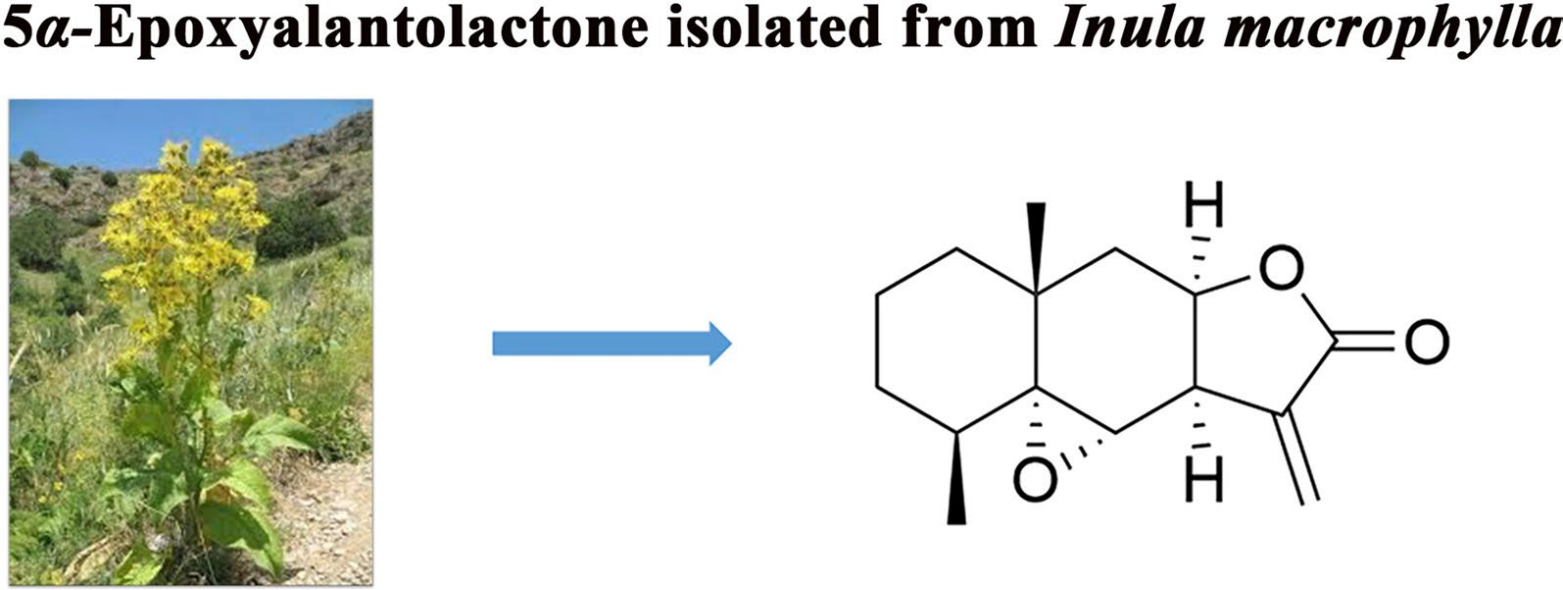
Chemical structures of natural product 5*α*-EAL

**Scheme 1:**
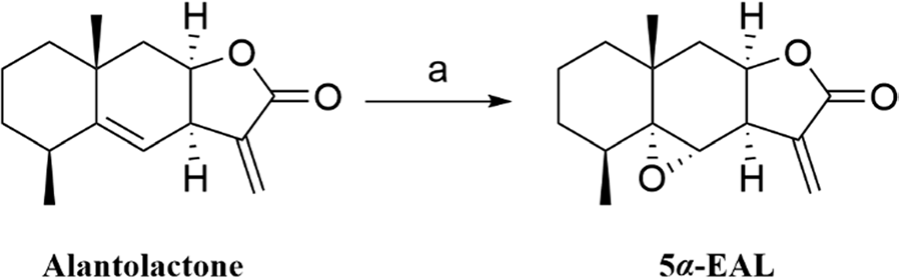
Synthesis of 5*α*-EAL by epoxidation of alantolactone. Reagents and condition: (a) *m*-CPBA, CH_2_Cl_2_, rt, 87%

The ethanol extract of the whole of *Inula macrophylla* was separated and purified through silica gel column chromatography and preparative HPLC to yield 5*α*-EAL (Fig. 1, ~ 0.07%). Its structure was elucidated by a comprehensive analysis of spectroscopic data (HR-ESI–MS, ^1^H NMR, and ^13^C-NMR) and verified as reported in the literature [17]. The structural difference between alantolactone and 5*α*-EAL is confined to the C-5 and C-6 epoxide ring. Then 5*α*-EAL could be synthesized from alantolactone through epoxidation via the Prilezhaev Reaction (Scheme 1), which is beneficial for the subsequent animal experiments. The structure of synthesized 5*α*-EAL was further confirmed by comparison of 1D NMR with isolated 5*α*-EAL (Fig. S3). The purity of 5*α*-EAL was determined to be 98.1% by using HPLC (Fig. S5).

### Preliminary screening of NO inhibition in LPS-induced BV-2 cells

**Fig. 2:**
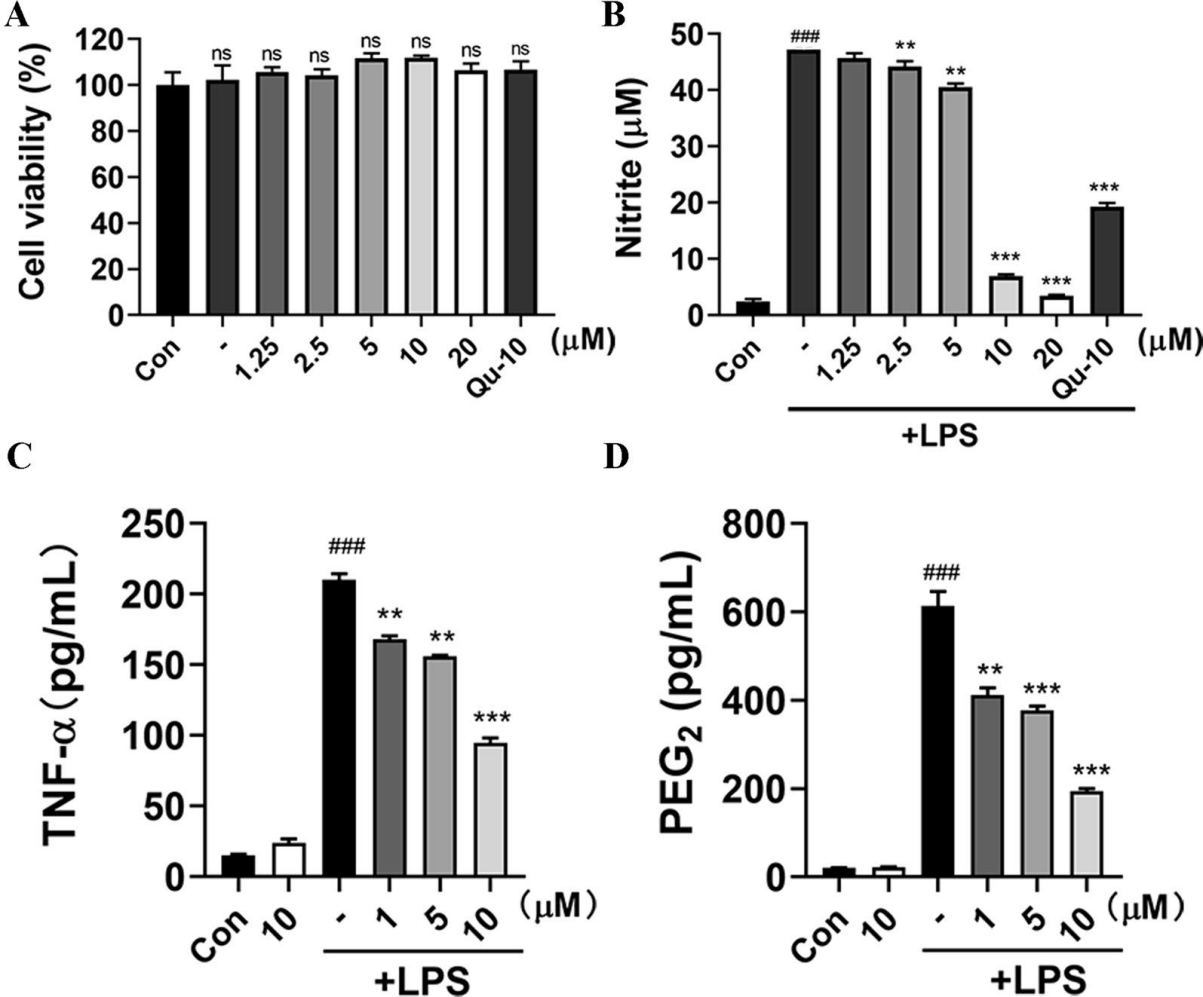
Effects of 5*α*-EAL on **A** BV-2 cell viability, **B** NO, **C** TNF-*α,* and **D** PGE_2_ production in BV-2 microglial cells induced by LPS. Cells were stimulated with or without 1 mg/mL LPS and treated with increasing concentration of 5*α*-EAL for 24 h. All values are the mean ± SEM of three independent experiments. ^###^*p* < 0.001 significantly different from the control group and **p < 0.01 and ***p < 0.001 vs LPS-treated control group. ns: not significant

Utilizing LPS-induced BV-2 cells as a model for neuroinflammatory cells, our previous work screened secondary metabolites isolated from *Inula macrophylla* at a 20 μM concentration, in which 5*α*-EAL exhibited a potential NO inhibition effect [16]. This study aims to further assess the level of NO production in microglia BV-2 induced by LPS, to estimate the anti-neuroinflammatory activity of 5*α*-EAL. First, the viability of BV-2 cells was assessed after treatment with different concentrations of the 5*α*-EAL (1.25, 2.5, 5, 10, and 20 μM). The findings indicated that 5*α*-EAL had no cytotoxicity towards BV-2 cells within the concentration ranging from 1.25 to 20 μM (Fig. 2A). The impact of 5*α*-EAL on NO production in BV-2 microglial cells was investigated in cells pre-treated and activated with LPS (1 μg/mL). In comparison to the control group (2.43 ± 1.08 pg/mL), stimulation of BV-2 cells with LPS led to a substantial increase in NO production (47.21 ± 1.97 pg/mL), and the treatment of 5*α*-EAL at different concentrations dose-dependently inhibited NO production (Fig. 2B). The NO levels were dramatically decreased to 6.93 ± 0.87 and 3.42 ± 0.53 pg/mL at 10 and 20 μM of 5*α*-EAL, respectively, which showed stronger inhibitory effect than that of positive control group (Quercetin, Qu) at 10 μM (19.26 ± 1.90 pg/mL). The EC_50_ value of 5*α*-EAL was calculated and determined to be 6.2 μM.

### 5*α*-EAL inhibits the production of TNF-*α* and PGE_2_ in LPS-stimulated BV-2 cells

Microglia serve as the primary defense mechanism for the central nervous system (CNS), functioning for resident macrophages within the brain, they exhibit two different phenotypes: pro-inflammatory M1 and anti-inflammatory M2 [18]. TNF-α and PGE_2_ are typical neuroinflammatory factors produced by M1 phenotypes of Microglia, which can cause neuroinflammation in the brain, and the production of TNF-α and PGE_2_ is essential for the synergistic generation of NO in LPS-stimulated BV-2 cells [19]. The effects of 5*α*-EAL treatments on TNF-α and PGE_2_ levels in LPS-stimulated BV-2 cells were evaluated. As depicted in Fig. 2C and D, at various concentrations (1, 5 and 10 μM), the production of TNF-α and PGE_2_ in LPS-stimulated BV-2 cells was significantly suppressed by 5*α*-EAL in a dose-dependent manner, and exhibited the highest inhibition effect at 10 μM among tested concentrations with the levels of TNF-α and PGE_2_ decreased to 94.77 ± 4.82 and 194.33 ± 8.39 pg/mL, respectively. The above result indicated that 5*α*-EAL can effectively inhibit the expression of proinflammatory cytokines TNF-α and PGE_2_.

### 5*α*-EAL inhibited the expression of iNOS and COX-2 in the LPS-stimulated BV-2 cells

**Fig. 3:**
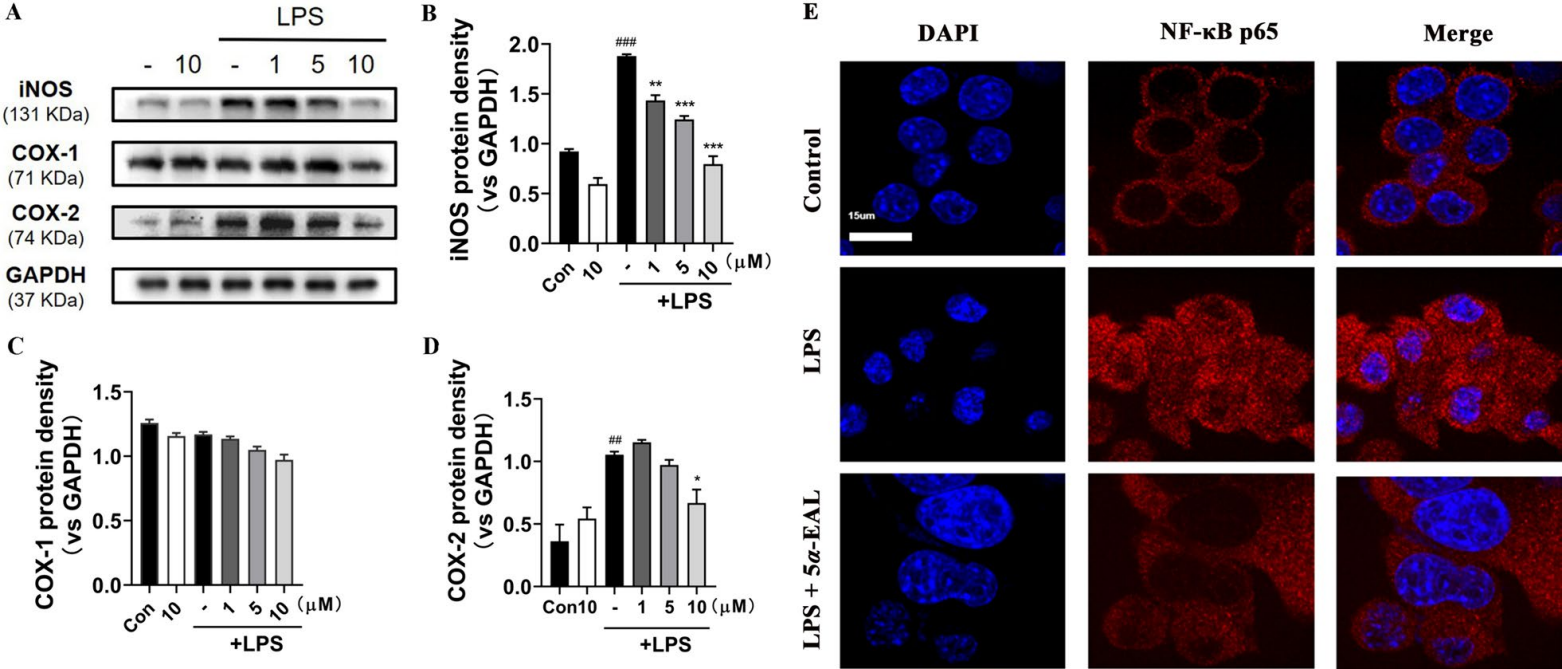
Inhibitory effects of 5*α*-EAL on iNOS, COX-1, and COX-2 production induced by LPS in BV-2 microglial cells. Cells were stimulated with or without 1 mg/mL LPS and treated with increasing concentrations of 5*α*-EAL for 24 h. **A** The iNOS, COX-1, and COX-2 protein expressions in BV-2 cells were determined by western blotting. Densitometric analyses of the **B** iNOS, **C** COX-1, and **D** COX-2. All values are the mean ± SEM of three independent experiments. ^##^*p* < 0.01, ^###^*p* < 0.001 significantly different from the control group and *p < 0.05 and **p < 0.01 and ***p < 0.001vs LPS-treated control group. **E** Effects of 5*α*-EAL at 10 μM on the translocation of NF-κB p65 in LPS-stimulated BV-2 cells. NF-κB p65 (red), DAPI (blue), Scale bar = 15 μM. Images were captured by confocal fluorescence microscope with ×100 objective

iNOS is an important enzyme for controlling the production of NO in LPS-simulated BV-2 cells. Increased production of NO through activation of iNOS expression leads to neuronal degeneration [20]. Based on the significant decrease in NO levels after the administration of 5*α*-EAL, the expression of iNOS was further studied by Western blot analysis (Fig. 3A and B). The iNOS expression level in LPS-simulated cells was significantly higher than control and 5*α*-EAL at 10 μM group (*p* < 0.001) without LPS treatment. 5*α*-EAL pretreatment at the different concentrations (1, 5 and 10 μM) remarkably inhibited iNOS expression in a concentration-dependent manner. 5*α*-EAL exhibited the highest inhibition effect (0.79 ± 0.11, *p* < 0.001) at 10 μM among tested concentrations and still exhibited a statistically significant disparity in comparison to the LPS-treated group (1.43 ± 0.08, *p* < 0.01) at 1.0 μM. The protein expressions of cyclooxygenase-1 (COX-1) and cyclooxygenase-2 (COX-2) which are responsible for the production of PGE_2_ were further investigated (Fig. 3A, C, and D). 5*α*-EAL dose-dependently reduces the COX-2 expression level while almost having no effect on the expression of COX-1 within the range of test concentrations. Meanwhile, the expression level of COX-2 in BV-2 cells without LPS treatment was significantly lower than that of COX-1. These data demonstrated that 5*α*-EAL could regulate NO and PGE_2_ production by inhibiting the expression of iNOS and COX-2. Nuclear factor-kappa-B (NF-κB) is one of a key factor in the expression of iNOS and COX-2, which is mediated by LPS-stimulated inflammatory mediators [21]. To confirm the blockade effect of 5*α*-EAL on NF-κB p65 activity, the nuclear translocation of NF-κB p65 was evaluated by using immunofluorescence assay. The results revealed that 10 μM of 5*α*-EAL treatment effectively reduced LPS-induced nuclear translocation of NF-κB p65 (Fig. 3E). This result suggests that the inhibition of NF-κB activation by 5*α*-EAL may be the key signaling pathway responsible for suppressing iNOS, COX-2, and other proinflammatory cytokines in LPS-stimulated BV-2 cells.

### 5*α*-EAL inhibited the production of IL-10 in LPS-stimulated BV-2 cells

**Fig. 4:**
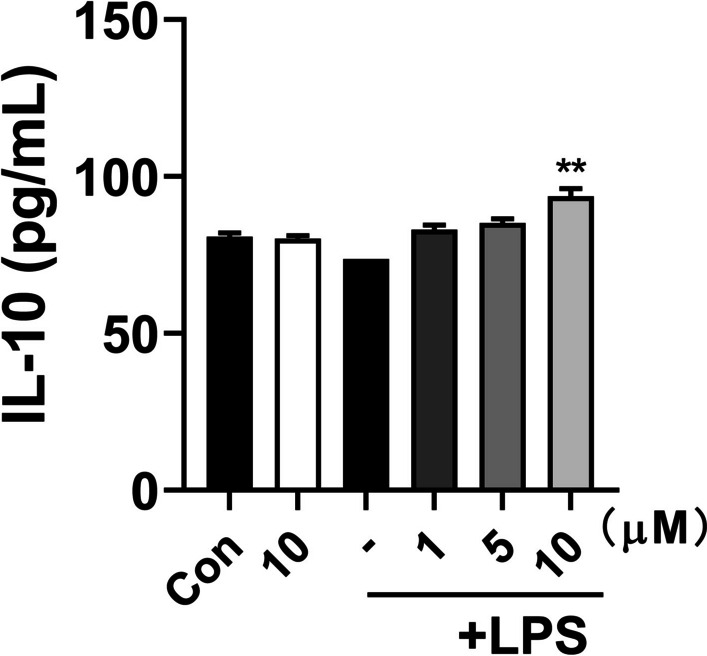
Inhibitory effects of 5*α*-EAL on IL-10 production induced by LPS in BV-2 microglial cells. Cells were stimulated with or without 1 mg/mL LPS and treated with increasing concentrations of 5*α*-EAL for 24 h. All values are the mean ± SEM of three independent experiments. **p < 0.01 vs LPS-treated control group

In contrast to the M1 phenotype, the anti-inflammatory M2 phenotype of microglia can inhibit the inflammatory response, and facilitate the transition of macrophage polarization from the M1 phenotype to the M2 phenotype, which helps exert a neuroprotection effect in the treatment of CNS-related disease [18]. To investigate whether 5*α*-EAL affects the BV-2 cell polarization, the representative M2 phenotype markers, IL-10, were evaluated. As shown in Fig. 4, after treatment with LPS, the M2 pro-inflammatory cytokine IL-10 level did not show significant changes in BV-2 cells. Furthermore, 5*α*-EAL significantly increased the production of IL-10 (93.67 ± 3.54 pg/mL, *p* < 0.01) at a concentration of 10 μM. The above results indicated that 5*α*-EAL can facilitate the conversion process of microglia phenotype from the pro-inflammatory M1 phenotype to the anti-inflammatory M2 phenotype.

### Capacity of 5*α*-EAL in penetrating blood–brain barriers (BBB)

In the process of developing CNS drugs, it is essential to assess and predict the BBB permeability of candidate compounds. The parallel artificial membrane permeability assay (PAMPA) is a high-throughput technique that has been extensively used for predicting BBB permeability [22]. The permeability (*P*e) of 5*α*-EAL and positive drugs (carbamazepine and hydrocortisone) was quantified, following the previously reported method [23]. The results presented in Table 1 demonstrate that the *P*e values of the two positive drugs are in line with the previously reported findings, indicating the reliability of the assay employed in this study. According to these ranges, it was found that 5*α*-EAL is capable of crossing the BBB (*P*e (10^−6^ cm/s) = 6.45), which is beneficial for the management of neurodegenerative diseases.

**Table 1:** Permeability results of 5*α*-EAL and control drugs from the PAMPA-BBB assay

Compounds	*P*_e_ (× 10^–6^ cm/s)^a^	CNS prediction
5*α*-EAL	6.45	+
Carbamazepine	10.8	+
Hydrocortisone	1.9	−

^a^Compound was dissolved in DMSO at 8 mg/mL, then diluted with PBS. Compounds with permeabilities *P*_e_ > 4.7 × 10^–6^ cm s^−1^ could cross the BBB by passive diffusion. Pe (10^−6^ cm/s) > 4.0, “CNS+” (high BBB permeation); Pe (10^−6^ cm/s) < 2.0, “CNS−” (low BBB permeation)

### 5*α*-EAL ameliorates the cognitive dysfunction of mice induced by scopolamine in the behavioral tests

**Fig. 5:**
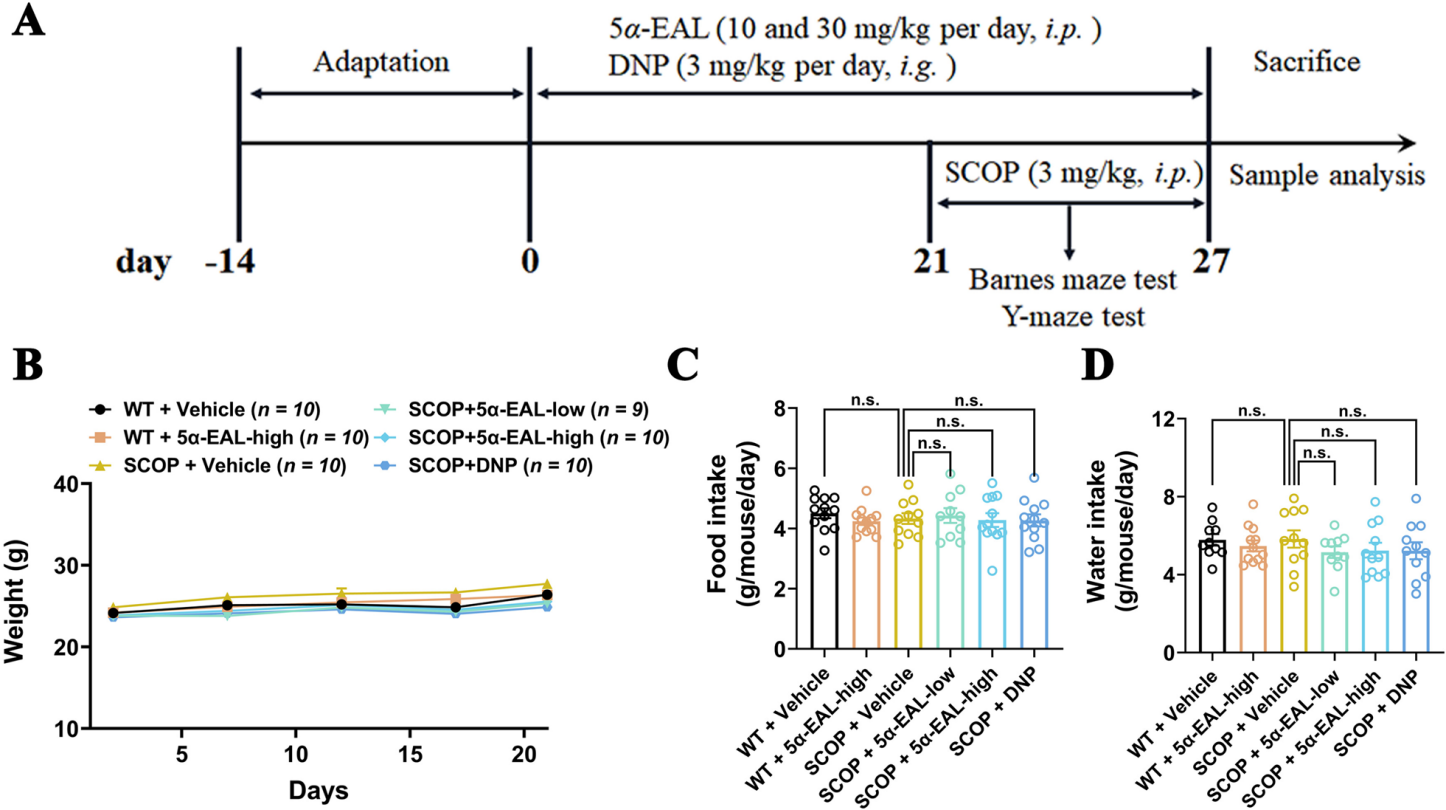
Timeline and the effects of 5*α*-EAL supplementation on mice’s body weight, food intake, and water intake. **A** Overall experimental schedule. **B** Change of the body weight of mice with time. There is no significant difference in the weight of all mice in the period. The food (**C**) and water (**D**) intakes of mice in each group, respectively. No significant difference is among all groups. Data are means ± SEM, n.s., not significant; one-way analysis of variance (ANOVA) followed by Tukey multiple comparisons test

The muscarinic antagonist scopolamine (SCOP)-induced amnesia model is a widely used animal model to induce amnesia in mammals [24]. Spatial learning and memory are known to be impacted by the cognitive deficits which associated with the progression of AD. The activity of 5*α*-EAL on the ameliorative effects against cognitive dysfunction in the SCOP-induced AD mice model was further investigated, and donepezil (DNP) served as a positive control drug. The trial schedule is detailed in Fig. 5A. The body weight, food, and water intake were evaluated in WT and SCOP-induced model mice that received either vehicle or 5*α*-EAL treatment to examine the potentially toxic effects of 5*α*-EAL on the mice. As shown in Fig. 5B–D, the body weight of mice after treatment with 5*α*-EAL did not differ from that of the WT group (WT + Vehicle, 25.15 ± 1.79 g; WT + 5*α*-EAL-high, 25.38 ± 1.27 g; SCOP + Vehicle, 26.35 ± 1.86 g; SCOP + 5*α*-EAL-low, 24.42 ± 1.81 g; SCOP + 5*α*-EAL-high, 24.88 ± 1.78 g; SCOP + DNP, 24.26 ± 1.21 g). Furthermore, there were no significant differences in dietary and water intake observed among these groups. The results suggest that the administration of 5*α*-EAL is not toxic to mice.

#### 5*α*-EAL ameliorates the spatial memory impairment in the Barnes maze task

**Fig. 6:**
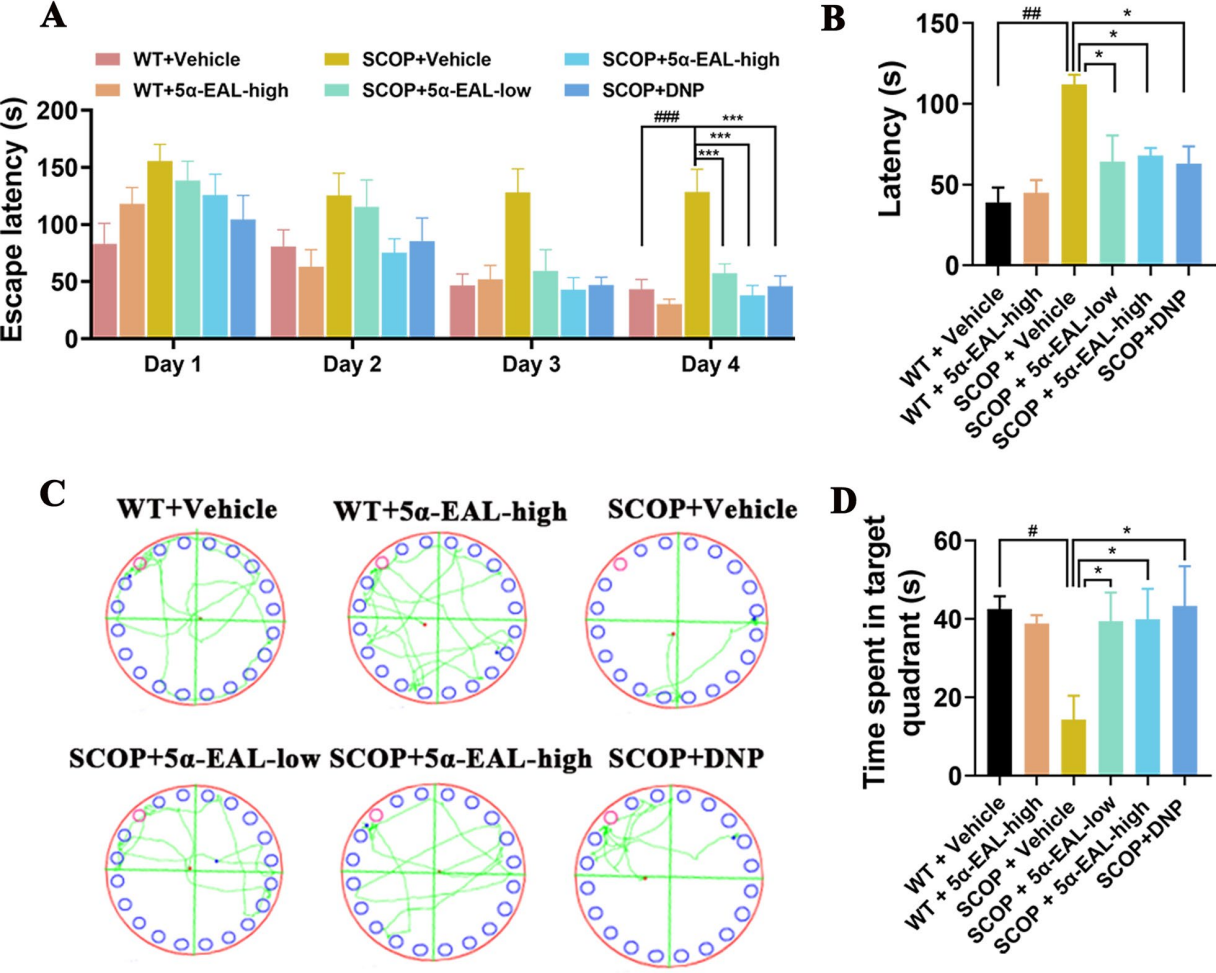
Effects of 5*α*-EAL on scopolamine-induced cognitive dysfunction based on the Barens maze test results in scopolamine-induced AD mice. **A** The escape latency of four consecutive days test. **B** The escape latency of day 5. **C** Barens maze path of day 5 and **D** time spent in the target quadrant were recorded in the Barens maze test. Data are expressed as the mean ± SEM (n = 9–10). ^##^*p* < 0.01and ^#^*p* < 0.05 vs. the WT + Vehicle group. *p < 0.05 and ***p < 0.001vs. the SCOP + Vehicle. WT + Vehicle: Control group; SCOP + Vehicle: Scopolamine group; SCOP + 5*α*-EAL-low: Scopolamine + 10 mg/kg 5*α*-EAL group; SCOP + 5*α*-EAL-high: Scopolamine + 30 mg/kg 5*α*-EAL group; SCOP + DNP: Scopolamine + Donepezil group

The Barnes maze was designed to evaluate the visuospatial learning and memory of SCOP-induced mice in behavioral tests. The escape latency has no obvious differences on days 1 and 2 but exhibited significant differences on days 3 and 4. At day 4, the escape latency of the mice induced by SCOP with 5*α*-EAL treatment (SCOP + 5*α*-EAL-low, 57.35 ± 23.43 s; SCOP + 5*α*-EAL-high, 38.04 ± 26.18 s) was significantly reduced compared to the SCOP + Vehicle group (128.49 ± 56.45 s) (Fig. 6A). Moreover, the SCOP + Vehicle group exhibited a longer escape latency compared to the group of WT + Vehicle (43.36 ± 25.56 s), demonstrating that mice induced by SCOP caused cognitive impairments and spatial memory loss. In the spatial exploration experiment, the escape latency and characteristic traces on day 5 of mice are shown in Fig. 6B and C. The group of SCOP + 5*α*-EAL (SCOP + 5*α*-EAL-low, 64.31 ± 45.74 s; SCOP + 5*α*-EAL-high, 67.96 ± 12.47 s) spent a shorter period in the target quadrant in comparison to the SCOP + Vehicle group (112.03 ± 15.95 s), which exhibited similar performances in the SCOP + DON group (63.09 ± 30.12 s). As shown in Fig. 6D, the time proportion in the group of SCOP + Vehicle (14.37 ± 17.09 s) was considerably below that in the WT + Vehicle group (42.53 ± 10.43 s), after the treatment of 5*α*-EAL in SCOP-induce mice, the time proportion was significantly increased (SCOP + 5*α*-EAL-low, 39.49 ± 21.79 s; SCOP + 5*α*-EAL-high, 39.98 ± 21.96 s). These results indicate that 5*α*-EAL can ameliorate cognitive impairment in AD mice.

#### 5*α*-EAL ameliorates the working memory impairment in the Y-maze task

**Fig. 7:**
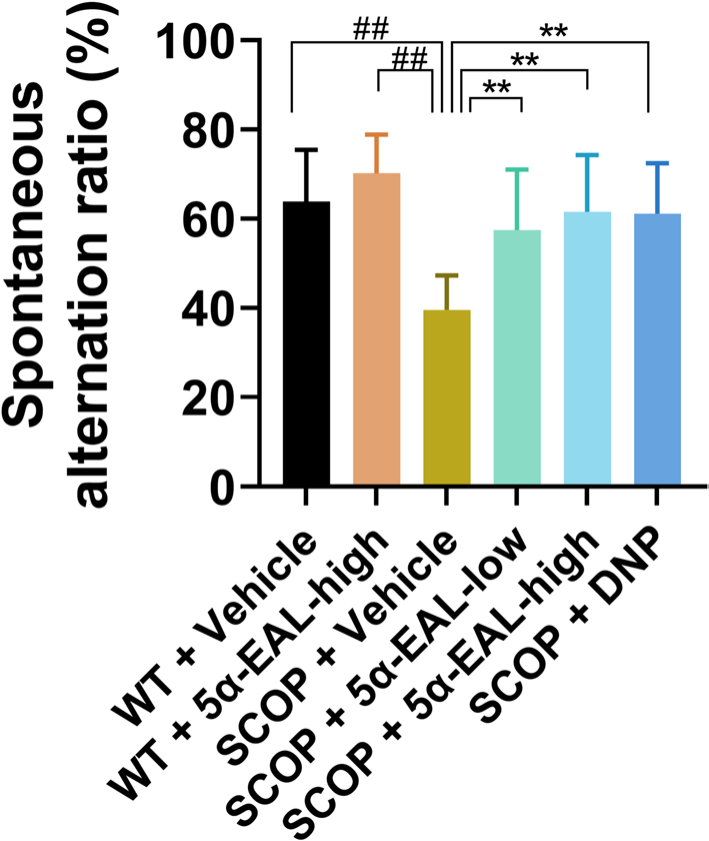
Effects of 5*α*-EAL on scopolamine-induced cognitive dysfunction based on spontaneous alternation ratio in a Y-maze test. Data are expressed as the mean ± SEM (n = 9–10). ^##^*p* < 0.01 vs. the WT + Vehicle group. **p < 0.01 vs. the SCOP + Vehicle

The Y-maze was used to examine the effect of 5*α*-EAL on spontaneous alternation behavior. The findings indicated that the group of SCOP + Vehicle (39.57 ± 7.23 s) exhibited a significantly lower percentage of spontaneous alterations in comparison with the WT + Vehicle group (63.91 ± 11.56 s) (Fig. 7). On the contrary, treatment with 5*α*-EAL (SCOP + 5*α*-EAL-low, 57.42 ± 13.63 s; SCOP + 5*α*-EAL-high, 61.55 ± 12.75 s) significantly increase the percentage of spontaneous alternation in comparison to the group of SCOP + Vehicle, indicating that the administration of 5*α*-EAL could mitigate the working memory impairment of mice after treated by SCOP.

### Effect of 5*α*-EAL on AChE activity in the hippocampus and cortex

**Fig. 8:**
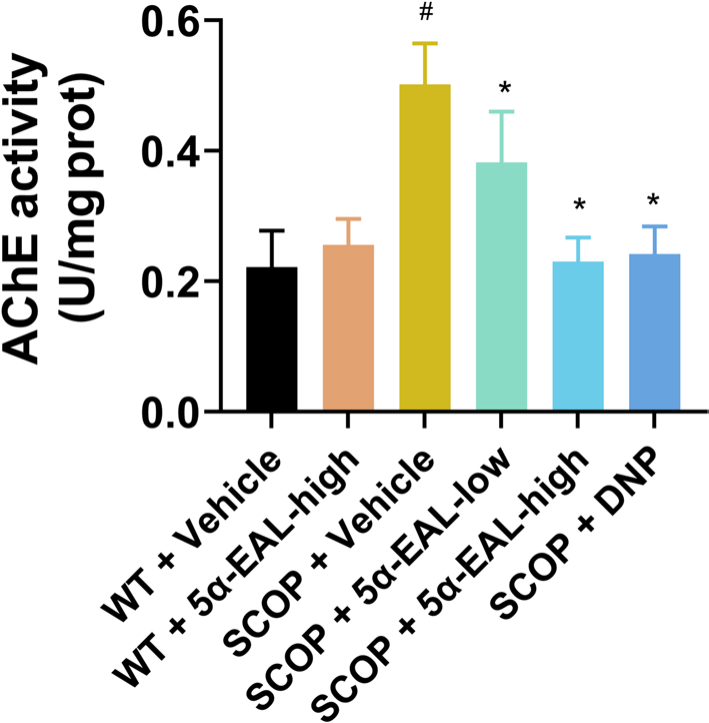
Effect of 5*α*-EAL on AChE activity in the hippocampus and cortex of the scopolamine-induced mice. The activities of AChE in the brain were measured by assay kits. Data are expressed as the mean ± SEM (n = 9–10). ^#^*p* < 0.05 vs. the WT + Vehicle group and *p < 0.05 vs. the SCOP + Vehicle group

AChE is a cholinergic enzyme in charge of breaking down acetylcholine [25], which is a key enzyme in the cholinergic system. The AChE activity in cortex and hippocampus after 5*α*-EAL treatment was investigated and the results was depicted in Fig. 8. The activity of AChE was substantially elevated in the SCOP + Vehicle group (0.50 ± 0.08 U/mg prot) in comparison with the WT + Vehicle group (0.22 ± 0.15 U/mg port). Whereas the group of SCOP + 5*α*-EAL-high group (0.23 ± 0.05 U/mg prot) could significantly decrease the activity of AChE in comparison with the SCOP + Vehicle group (0.50 ± 0.10 U/mg prot). Briefly, 5*α*-EAL has the potential to mitigate the cholinergic system imbalance in SCOP-induced mice.

## Conclusion

In summary, it was found that 5*α*-EAL could significantly reduce NO, TNF-α, and PGE_2_ production by inhibiting iNOS and COX-2 expressions in LPS-stimulated BV-2 cells. 5*α*-EAL modulates the transformation of M1 phenotype macrophages to M2 phenotype macrophages after LPS stimulation in vitro*,* as well as penetrating the BBB. Additionally, 5*α*-EAL (10 and 30 mg/kg per day, *i.p.*) significantly improves cognitive impairments of AD mice in behavioral tests including Barnes and Y-maze tests, and 5*α*-EAL could reduce the activity of AChE in the hippocampus and cortex of AD mice. These findings confirmed that the 5*α*-EAL may be a promising natural product worthy of further exploitation for AD therapy.

## Experimental section

### Chemistry

#### Isolation and identification of 5*α*-EAL from *Inula macrophylla*

Whole plant collections are from Uzbekistan, in 2021 and were recognized as *Inula macrophylla* by Dr. K. A. Eshbakova. The specimen of the voucher was archived at the Academy of Sciences of the Republic of Uzbekistan.

10.0 kg of the *Inula macrophylla* were air-dried, powdered, and extracted with 90% ethanol. After evaporation, the residue was suspended in H_2_O and successively partitioned with EtOAc. The EtOAc layer (308 g) was fractionated into five fractions (A‒E) via silica gel column chromatography eluting with CHCl_3_–MeOH (from 60:1 to 3:1, v/v). Fr. B (18 g) was fractionated using medium-pressure liquid chromatography with a MeOH–H_2_O mobile phase (from 10:90 to 90:10, v/v) over a period of 50 min, resulting in the isolation of five fractions. Fr. B2 was purified by preparative HPLC (MeCN–H_2_O from 40:60 to 60:40, v/v, 60 min) to obtain 5*α*-EAL. The 1D NMR spectra were detected via a Bruker Avance III 400 instrument (Figs. S1 and S2).

### In vitro activity

#### Cell viability assay in BV-2 cells

Murine microglial BV-2 cells were plated at a concentration of 8 × 10^3^ cells/well in 96-well plates and cultured overnight. After treatment with different levels of 5*α*-EAL (1.25, 2.5, 5, 10 and 20 µM) for 24 h, 100 µL of MTT solution (5 mg/mL) from Solarbio in China was pipetted into each well. The plates were then incubated at 37 °C for 2 h. The blue formazan products in the cells were diluted in DMSO (200 µL/well) and mixed thoroughly. The optical density of the reaction medium was read at a wavelength of 570 nm using a microplate reader.

#### Inhibition of nitric oxide production in BV-2 cells

NO production inhibitory was measured according to the previous method [8]. Briefly, 96-well plates were plated with murine microglial BV-2 cells (2 × 10^5^/well) and treated for 24 h with or without 1 μg/mL LPS (Escherichia coli 0111: B4, Sigma, U.S.) by adding 5*α*-EAL. The NO concentration in the supernatant was determined with an NO content assay kit (Solarbio, China).

#### ELISA assay

After treatment of LPS-stimulated BV-2 cells with 5*α*-EAL for 24 h, pro-inflammatory (TNF-α, PGE_2_) and anti-inflammatory (IL-10) mediator levels in culture supernatants were measured using ELISA kits according to the manufacturer’s method. (TNF-α: SU-BN20852, IL-10: SU-BN20162, R&D, USA; PGE_2_: KA0326, Abnova, China, Arg-1: ab269541, Abcam, USA).

#### Western blotting assay

The western blotting assays were measured according to the previous method [26]. In short, BV-2 cells were either exposed to 1 μg/mL LPS or left untreated, followed by treatment with DMSO or 5*α*-EAL at various concentrations. After 24 h, the cells were rinsed with chilled PBS (Solarbio). The chilled lysis buffer containing protease inhibitor and phosphatase inhibitor mixture (AbMole BioScience, China) was added to the cells and then gently scratched off. After centrifuging at 12,000 rpm for 10 min at 4 °C, Protein concentrations were assessed utilizing a bicinchoninic acid (BCA) assay kit from TIANGEN, China. Equivalent quantities of protein were subjected to electrophoresis on 10% sodium dodecyl sulfate-polyacrylamide gels from EpiZyme, China, and subsequently transferred to NC membranes manufactured by GVS, USA. Following this, the membranes underwent blocking with 5% nonfat milk from BD for 1 h before being incubated overnight at 4 °C with primary antibody diluted appropriately. Subsequent to washing, the membranes were exposed to a specific secondary antibody for a duration of 2 h. The chemiluminescence western blotting detection system (Thermo Fisher Scientific, USA) was used to visualize the immunoreactive bands. ImageJ software was then used for band analysis. The main primary antibodies used in this study were rabbit monoclonal anti-iNOS (1:1000, Abcam, USA), rabbit monoclonal anti-COX-1 (1:1000, CST, USA), rabbit monoclonal anti-COX-2 (1:1000, CST, USA) and rabbit monoclonal anti-GAPDH (1:1000, CST). The secondary HRP-conjugated antibody was goat anti-rabbit IgG (1:5000, SAB).

#### Immunofluorescence for NF-κB translocation assay

Immunofluorescence experiment was conducted according to the previous method to evaluate the nuclear translocation of NF-κB p65 [26]. In brief, the BV-2 cells were plated in 8-well plates and incubated for 24 h. The cells were pretreated with 5*α*-EAL (10 μM) for 1 h and then activated by LPS (1 μg/mL). Then, the fixed cells were permeabilized with 0.2% (v/v) Triton X-100 for 10 min in 4 °C and washed three times with PBS containing 5% BSA and blocked with 5% bovine serum albumin (BSA in PBS) for 1 h at room temperature. The permeabilized cells were incubated with primary antibody (anti-p-NF-κB p65) overnight at 4 °C. After removing the primary antibody, the cells were incubated with Anti-rabbit IgG (H+L), F (ab′)2 Fragment (Alexa Fluor® 647 Conjugate) for 1 h at room temperature. After removing the secondary antibody, cells were stained with 4′,6-diamidino-2-phenylindole (DAPI, 5 μg/mL in PBS) for 10 min. All images were obtained using a fluorescence microscope (Leica German).

#### Blood–brain barrier permeability assay

The BBB permeability of 5*α*-EAL was assessed using the parallel artificial membrane permeation assay (PAMPA) method, as previously described. The pre-coated PAMPA plate system (Cat. No. 353015) from Coning, U.S., was used to perform the permeability assays. Carbamazepine and hydrocortisone were supplied by Sigma. 5*α*-EAL was diluted in DMSO at a concentration of 8 mg/mL to form a stock solution, which was then diluted in PBS to a final concentration of 400 μg/mL. The solution was filtered through 0.22 μM syringe filters and pipetted into the donor wells (300 μL/well), while PBS was added to the wells of the recipient plate (200 μL/well). The filter plate was then assembled with the acceptor plate, and the entire assembly was incubated at room temperature for 16 h without agitation. At the end of the incubation, 150 μL of supernatant was removed from the donor and acceptor well and quantified by HPLC with a UV screening detector at 210 nm.

### In vivo activity

#### Animals and ethical statement

Male C57BL/6J mice were obtained from the Fourth Military Medical University, Shaanxi, China (n = 10/group) at 6–8 weeks of age and 25–30 g in weight. Animals were placed in a dedicated germ-free animal room under conditions of 12/12 light/dark cyclicity. They were provided with standardized nutrition, which consisted of pure water and AIN-93M. All animal procedures were performed under the principles and guidelines outlined in the Care and Use of Laboratory Animals.

In this study, all mice were randomized into six groups (n = 10/group) as shown below: group I, control (WT + Vehicle); group II, control + high dose 5*α*-EAL (30 mg/kg per day, WT + 5*α*-EAL-high); group III, SCOP (SCOP + Vehicle); group IV, SCOP + low-dose 5*α*-EAL (10 mg/kg per day, SCOP + 5*α*-EAL-low); group V, SCOP + high-dose 5*α*-EAL (30 mg/kg per day, SCOP + 5*α*-EAL-high); group VI, SCOP + DNP (3 mg/kg per day, SCOP + DNP). 5*α*-EAL (10 and 30 mg/kg per day) was dissolved in a solution of water containing 10% Tween 80, 10% ethanol, and 80% distilled water (0.1% w/vol). Donepezil Hydrochloride was dissolved in saline. The aforementioned groups were administered to 8-month-old WT mice for a period of 3 weeks. The WT + Vehicle, WT + 5*α*-EAL-high, SCOP + 5*α*-EAL-low, and SCOP + 5*α*-EAL-high groups received treatment via intraperitoneal (*i.p*.) injection of 5*α*-EAL, while the DNP group was treated with an oral gavage (*i.g.*) of DNP.

#### Barnes maze task

The Barnes Maze is the most widely used test in behavioral neuroscience to study spatial learning and memory in rodents. Briefly, mice were acclimated to the experimental room for 30 min before each daily experiment. Subsequently, they were placed in the center of the Barnes Maze in a starting box for 20 s. The start box was taken away and the mice were permitted to explore the Barnes maze for 3 min. If the mice did not locate the destination box on the first day, they were guided to find the hole and then remain in the box for 1 min. There was no guidance provided on days 2–4. Mice were under study for 4 consecutive days. On the fifth day, the dark box of the target hole was withdrawn, and the latency time, residence time in the destination quadrant, number of times of exploring the target hole, and the movement trajectory of the mice were recorded in the 90 s period. The table was wiped with 75% disinfectant alcohol before and after the test to remove odor interference.

#### Y-maze task

The Y-maze experiment is a common experimental tool for assessing working memory. The Y-maze comprises three equal arms (40 cm × 15 cm × 9 cm). Mice were placed in the center of the Y-maze and given 5 min to explore the three arms. Their motion was automatically recorded with a video tracker.

#### Measurement of AChE activity

After the behavior tests on day 28, Mice were executed by cervical dislocation. The mouse cortex and hippocampus were collected, washed with cold sterile saline, and stored at − 80 °C prior to use. The tissues of cortices and hippocampi were weighed, homogenized, and centrifuged at 4 °C 4000 rpm for 5 min. After 10 min collect the supernatant. The measurement of AChE activity was conducted following the instruction of acetylcholinesterase assay kit. This assay was conducted in a 2 mL centrifuge tube, adding 30 µL of 10% tissue solution, 500 µL of substrate buffer, and 500 µL of coloring liquid for each group. Incubate at 37 °C for 6 min, then add 30 µL of inhibitor and 100 µL of transparent agent, respectively. After incubation for 15 min, the OD value was measured at 412 nm using a microplate reader, and then activity was calculated.

#### Statistical analysis

GraphPad 8.0 software was used for statistical analysis. The experimental data were presented as mean ± SEM for each group. Student’s t-test or two-way analysis of variance (ANOVA) followed by Tukey post hoc test was used for all statistical analyses. *p < 0.05, **p < 0.01, and ***p < 0.001 were regarded as statistically significant for between-group differences.

### Supplementary Information

Supplementary Material 1. Synthesis of 5*α*-EAL by epoxidation of alantolactone; NMR spectrum, HR-ESI–MS spectrum, and HPLC spectra for 5*α*-EAL.

## Data Availability

All data generated or analyzed during this study are included in this published article and its supplementary information files.
