# Supplementary material for: 5α-Epoxyalantolactone from Inula macrophylla attenuates cognitive deficits in scopolamine-induced Alzheimer’s disease mice model
Source: Nat Prod Bioprospect. 2024 Jul 2;14(1):39. doi: 10.1007/s13659-024-00462-y (PMC11219692; doi:10.1007/s13659-024-00462-y)
Supplement: Supplementary file 1 — Supplementary Material 1. Synthesis of 5α-EAL by epoxidation of alantolactone; NMR spectrum, HR-ESI–MS spectrum, and HPLC spectra for 5α-EAL. [file 13659_2024_462_MOESM1_ESM.docx]

Supporting Information for

**5*α*-Epoxyalantolactone from *Inula macrophylla*** **attenuates** **cognitive deficits in scopolamine-induced Alzheimer’s disease mice model**

Rui Ma^a1^, Xu-Yao Feng^b1^, Jiang-Jiang Tang^b^, Wei Ha^a*^, Yan-Ping Shi^a*^

^a^ CAS Key Laboratory of Chemistry of Northwestern Plant Resources, Key Laboratory for Natural Medicines of Gansu Province, Lanzhou Institute of Chemical Physics, Chinese Academy of Sciences (CAS), Lanzhou 730000, P. R. China

^b^ Shaanxi Key Laboratory of Natural Products & Chemical Biology, College of Chemistry & Pharmacy, Northwest A&F University, No. 3 Taicheng Road, Yangling, Shaanxi, 712100, China

*Corresponding authors: hawei2012@licp.cas.cn (W Ha), shiyp@licp.cas.cn (Y.-P. Shi)

^1^: Rui Ma and Xu-Yao Feng contributed equally to this work.

**Table of** **Contents**

**Sections S1.** **Chemistry**

**S1.1** Chemistry

**S1.2** Spectroscopic data of 5*α*-EAL

**Sections S2. Supplementary of NMR, HR-ESI-MS, and HPLC**

**S2.1** ^1^H and ^13^C-NMR spectrum of 5*α*-EAL in CDCl_3_.

**S2.2** Compared to the 1D NMR of 5*α*-EAL in CDCl_3_.

**S2.3** HR-ESI-MS spectrum of 5*α*-EAL.

**S2.4** HPLC spectra of 5*α*-EAL.

**Sections S1. Chemistry**

**S1.1 Synthesis of 5*α*-EAL by** **epoxidation of alantolactone**

As shown in Scheme 1, the large amounts of 5*α*-EAL could be further synthesized from alantolactone according to a reported method.^1^ In brief, alantolactone (500 mg) was dissolved in dichloromethane (2.5 ml), and m-CPBA (525 mg) in dichloromethane (2.5 ml) was added dropwise to this solution. The mixture was stirred for 3 h at room temperature. Then the reaction mixture was diluted with 8 ml saturated Na_2_S_2_O_3_ solution and washed with saturated NaHCO_3_ solution. The organic layer was dried over anhydrous Na_2_SO_4_ and evaporated to afford a crude product, which was purified by preparative HPLC (Hanbon Sci & Tech of China Meger C18 Flow, 10 *µ*m, 20 × 250 mm, 205 nm, 220 nm, MeCN-H_2_O from 40:60 to 60:40, v/v, 25 min) to obtain 5α-EAL (435 mg, 87%).

**S1.2 Spectroscopic data of 5*α*-EAL**

White powder (purity: 98.1%). ^1^H NMR (400 MHz, CDCl_3_) *δ*_H_ (ppm) 6.42 (d, *J* = 2.9 Hz, 1H), 5.78 (d, *J* = 2.5 Hz, 1H), 4.68 (ddd, *J* = 8.9, 4.6, 1.9 Hz, 1H), 3.68 (m, 1H), 2.91 (d, *J* = 2.6 Hz, 1H), 1.89 (dd, *J* = 15.1, 4.5 Hz, 1H), 1.12 (s, 3H), 1.05 (d, *J* = 7.8 Hz, 3H). ^13^C NMR (100 MHz, CDCl_3_) *δ*_C_ 169.7, 136.7, 123.9, 75.2, 67.6, 61.2, 39.6, 37.7, 37.4, 37.1, 32.6, 29.6, 24.0, 18.1, 16.5. HR-ESI-MS *m/z* 271.1308 (M+Na)^+^ [calcd for C_15_H_22_O_2_Na^+^, 271.1305]. The purity of 5*α*-EAL was determined by using Agilent 1260 liquid chromatography system equipped with RedClassical C18 columns (5 µm, 1.6 × 250 mm, MeCN-H_2_O from 40:60 to 60:40, v/v, 15 min) and a diode array detector (DAD) (Agilent Technologies, Santa Clara, CA, US), the result seen in the supplementary figure (Fig. S5).

**Sections S2** Supplementary of NMR, HR-ESI-MS, and HPLC

**S2.1** ^1^H and ^13^C-NMR spectrum of 5*α*-EAL in CDCl_3_.


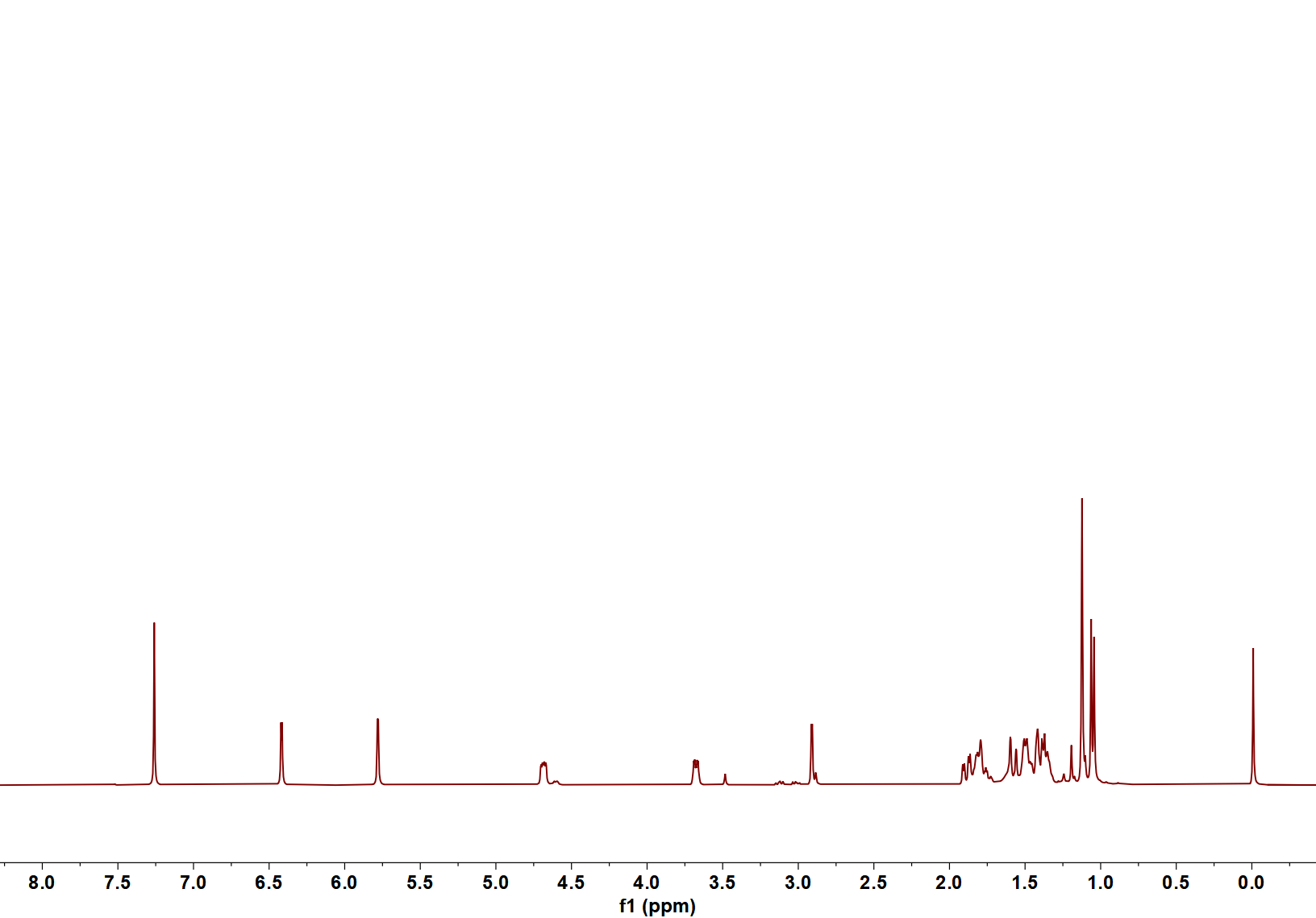


**Figure S1.** ^1^H-NMR spectrum of 5*α*-EAL in CDCl_3_.


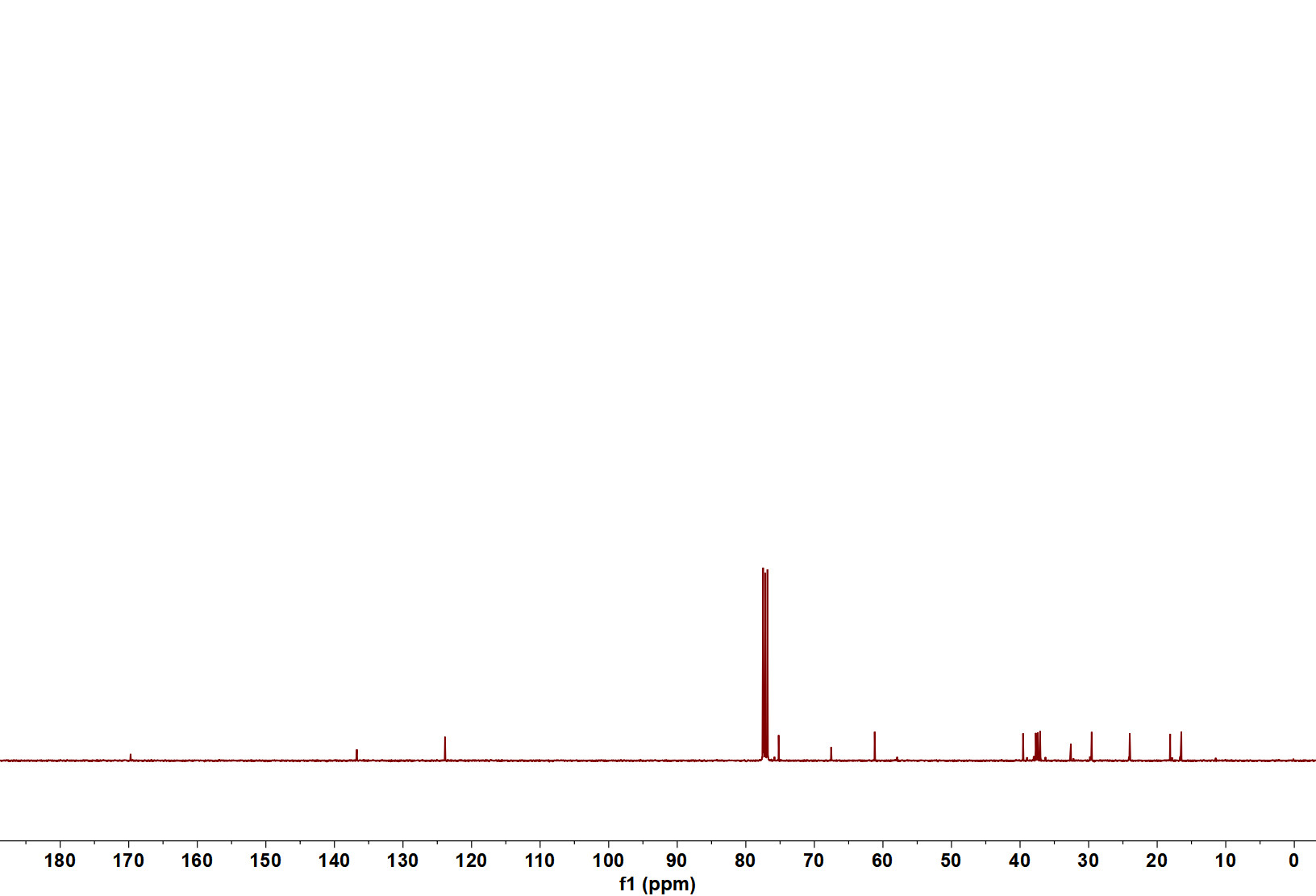


**Figure S2.** ^13^C-NMR spectrum of 5*α*-EAL in CDCl_3_.

**S2.2** Compared to the 1D NMR of 5*α*-EAL in CDCl_3_.


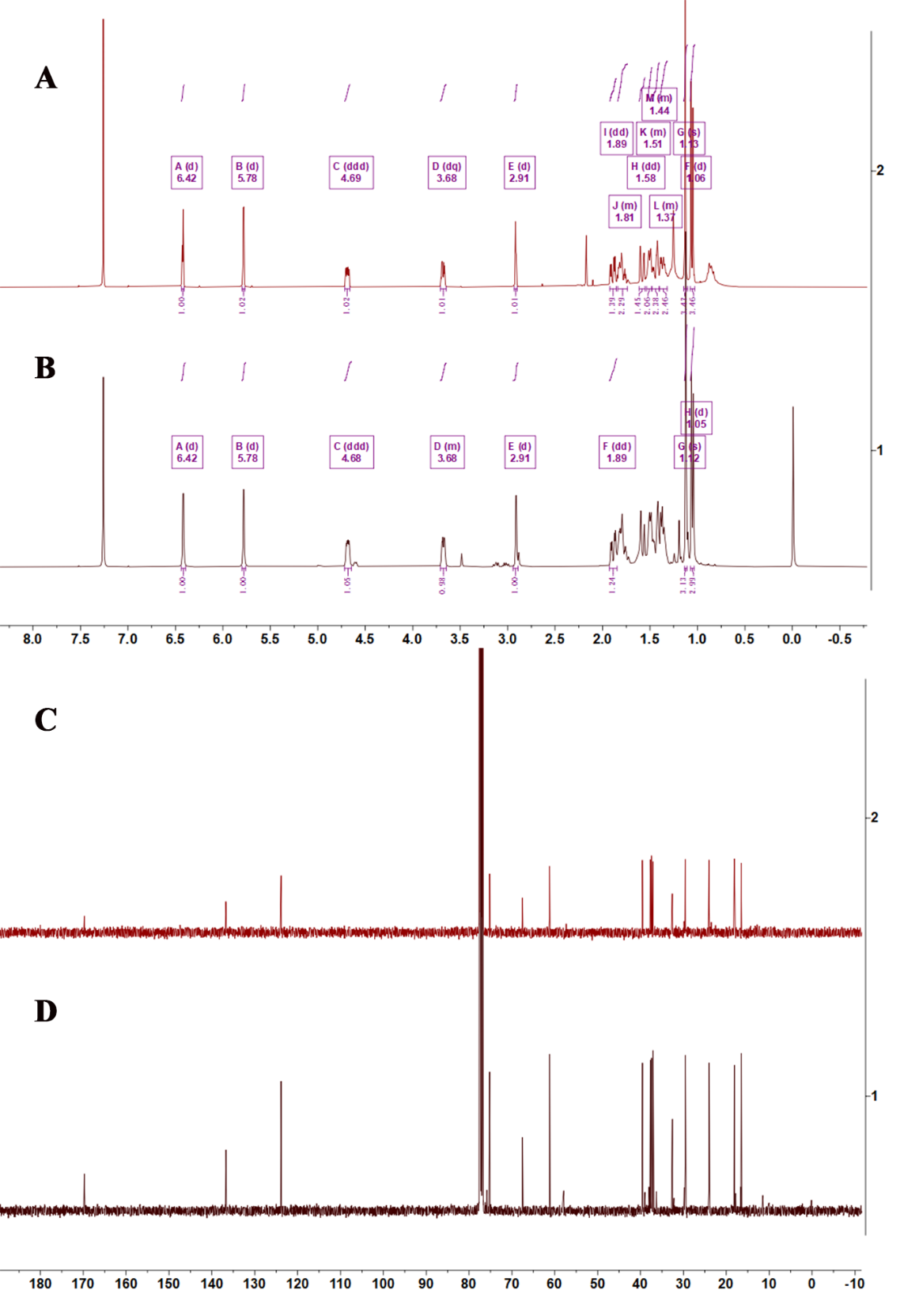


**Figure S3.** Compared to the 1D NMR of 5*α*-EAL in CDCl_3_.

(A) ^1^H-NMR of spectrum of synthesized product 5*α*-EAL; (B) ^1^H-NMR of spectrum of natural product 5*α*-EAL; (C) ^13^C-NMR of spectrum of Synthesized product 5*α*-EAL; (D) ^13^C-NMR of spectrum of natural product 5*α*-EAL;

**S2.3** HR-ESI-MS spectrum of 5*α*-EAL.


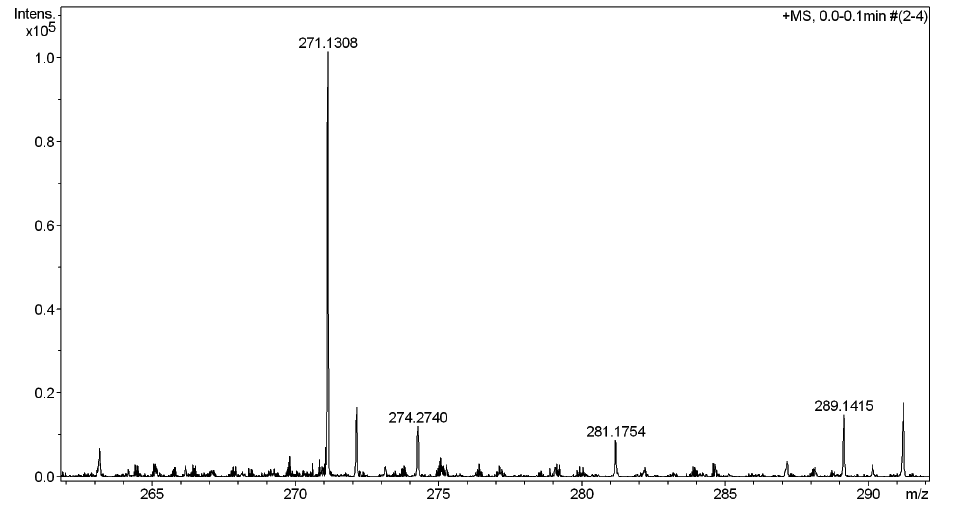


**Figure S4.** HR-ESI-MS spectrum of 5*α*-EAL.

**S2.4** HPLC spectra of 5*α*-EAL.


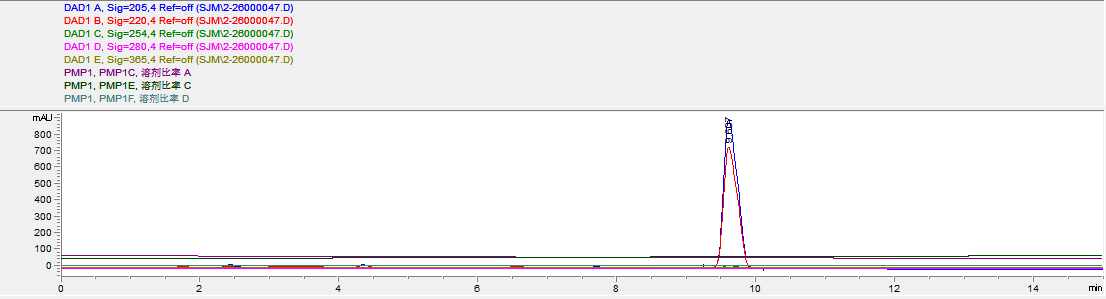


**Figure S5.** HPLC spectra of 5*α*-EAL.

References

1. Li X, Lu C, Liu S, et al. Synthesis and discovery of a drug candidate for treatment of idiopathic pulmonary fibrosis through inhibition of TGF-β1 pathway. *Eur. J. Med. Chem.* 2018; 157: 229-247. https://doi.org/10.1016/j.ejmech.2018.07.074.

2. Tang J-J, Huang L-F, Deng J-L, et al. Cognitive enhancement and neuroprotective effects of 1,6-O,O-diacetylbritannilactone, a sesquiterpene lactone in 5xFAD Alzheimer's disease mice model. Redox Biol. 2022; 50: 102229. https://doi.org/10.1016/j.redox.2022.102229.

3. Tang J-J, Wang M-R, Dong S, et al. 1,10-Seco-Eudesmane sesquiterpenoids as a new type of anti-neuroinflammatory agents by suppressing TLR4/NF-κB/MAPK pathways. *Eur J Med Chem.* 2021; 224: 113713. https://doi.org/10.1016/j.ejmech.2021.113713.
